# High serum bicarbonate level within the normal range prevents the progression of chronic kidney disease in elderly chronic kidney disease patients

**DOI:** 10.1186/1471-2369-14-4

**Published:** 2013-01-09

**Authors:** Eiichiro Kanda, Masumi Ai, Masayuki Yoshida, Renjiro Kuriyama, Tatsuo Shiigai

**Affiliations:** 1 Department of Nephrology, Tokyo Kyosai Hospital, Nakameguro 2-3-8, Meguroku, Tokyo, 153-8934, Japan; 2 Tokyo Medical and Dental University, Bioethics Research Center, Yushima 1-5-45, Bunkyoku, Tokyo, 113-8519, Japan; 3 Kokubunji Minamiguchi Clinic, Minamicho 3-15-6, Kokubunjishi, Tokyo, 185-0021, Japan; 4 Shiigai Clinic, Shinmachi 2-6-4, Torideshi, Ibaraki, 302-0024, Japan

**Keywords:** Bicarbonate, CKD, Elderly, CKD progression, Acidosis

## Abstract

**Background:**

Metabolic acidosis leads to chronic kidney disease (CKD) progression. The guidelines recommend a lower limit of serum bicarbonate level, but no upper limit. For serum bicarbonate level to be clinically useful as a therapeutic target marker, it is necessary to investigate the target serum bicarbonate level within the normal range to prevent CKD progression.

**Methods:**

One hundred and thirteen elderly CKD patients, whose serum bicarbonate level was controlled within the normal range, were enrolled in this retrospective cohort study in Ibaraki, Japan. Outcome was defined as a decrease of 25% or more in estimated glomerular filtration rate (eGFR) or starting dialysis. We used Cox proportional hazard models adjusted for patients’ characteristics to examine the association between serum bicarbonate level and the outcome.

**Results:**

Female patients were 36.3%: average age (SD), 70.4 (6.6) years; eGFR, 25.7 (13.6) ml/min/1.73 m^2^; serum bicarbonate level, 27.4 (3.2) mEq/l. Patients with the lowest quartile of serum bicarbonate levels [23.4 (1.8) mEq/l] showed a high risk of CKD progression compared with patients with high serum bicarbonate levels [28.8 (2.3) mEq/l]: adjusted hazard ratio (HR), 3.511 (95% CI, 1.342-9.186). A 1 mEq/l increase in serum bicarbonate level was associated with a low risk of CKD progression: adjusted HR, 0.791 [95% confidence interval (CI), 0.684-0.914].

**Conclusions:**

In elderly CKD patients, our findings suggest that serum bicarbonate level is independently associated with CKD progression, and that a high serum bicarbonate level is associated with a low risk of CKD progression. A high target serum bicarbonate level within the normal range may be effective for preventing CKD progression.

## Background

As the number of functioning nephrons decreases in chronic kidney disease (CKD), total ammonium excretion level begins to decrease when glomerular filtration rate (GFR) is below 40 to 50 mL/min/1.73 m^2^[1,2]. This results in net retention of hydrogen ions and metabolic acidosis [1,2]. Metabolic acidosis leads to CKD progression [3]. There is some evidence of the relationship between serum bicarbonate level and CKD progression. Observational studies showed an association between a low serum bicarbonate level and progression of kidney diseases [4-7]. Intervention studies show that bicarbonate supplementation slowed the progression of kidney diseases [8-12].

Kidney function declines with histological changes of the kidney with aging [13,14]. United States National Health and Nutrition Examination Surveys showed that a decreased GFR is associated with a high prevalence of acidosis in elderly CKD patients [15]. Although elderly CKD patients have a high risk of metabolic acidosis, we were unable to find any reports about the effect of serum bicarbonate level on CKD progression in elderly CKD patients.

 At present, the National Kidney Foundation Kidney Disease Outcomes Quality Initiative (K/DOQI) guidelines 2000 and the Care of Australians with Renal Impairment (CARI) guidelines recommend that serum bicarbonate level should be maintained at or above 22 mEq/l [16,17]. However, the guidelines recommend only this lower limit of the target serum bicarbonate level, but no upper limit in non-dialysis-dependent CKD patients. There have been no confirmatory controlled trials on the therapeutic range of serum bicarbonate levels. For serum bicarbonate level to be clinically useful, in this retrospective cohort study of elderly non-dialysis-dependent CKD patients whose serum bicarbonate levels were controlled within the normal range, we evaluated the relationship between serum bicarbonate level within the normal range and CKD progression, and investigated the upper limit of the target serum bicarbonate level.

## Subjects and methods

### Study design and study population

This study was a retrospective cohort study of non-dialysis-dependent CKD patients who were treated from 2009 to 2012 at Shiigai Clinic, Ibaraki, Japan. Patients were eligible for inclusion in the sample for this study when they were at least 60 years of age as of December 1st, 2009, diagnosed as having CKD on the basis of the criteria of the Japanese Society of Nephrology, had an estimated GFR (eGFR) of 60 ml/min/1.73 m^2^ or lower, had never been treated by dialysis or undergone transplantation, and their serum bicarbonate levels were within the normal range (normal range, 21 to 32 mEq/l) [18]. They usually visited the Shiigai Clinic once a month [mean interval of visit and standard deviation (SD), 33.5 ± 3.0 days]. Patients who were treated for dementia, lung diseases, chronic heart failure, or cancer were excluded. We treated CKD as a general practice of the clinic following the CKD practice guideline of the Japanese Society of Nephrology [18]. A high serum bicarbonate level was treated in accordance with K/DOQI guidelines 2000 [16]. Serum bicarbonate level was maintained from 22 to 32 mEq/l by administration of only sodium bicarbonate [16]. eGFR was calculated using the formula adopted by the Japanese Society of Nephrology using serum creatinine level [18]. This study was approved by the Ethics Committee of Tokyo Kyosai Hospital, Tokyo, Japan.

### Data

The baseline characteristics of the population were recorded at the time of the patients’ initial evaluation in the nephrology clinic. The patients’ demographics including age, gender, and history of diabetes mellitus and hypertension; laboratory variables, namely, albumin, sodium, potassium, creatinine, bicarbonate, and urinary protein levels; and the use of medications, namely, renin–angiotensin–aldosterone system (RAAS) inhibitors [angiotensin-converting enzyme inhibitor (ACEI), angiotensin II receptor blockers (ARBs) or direct renin inhibitors], loop diuretics, and sodium bicarbonate were obtained from the medical records of the patients treated at the clinic. Serum creatinine level was recorded longitudinally every month. Routine serum biochemistry was carried out by standard methods at Shiigai Clinic. Serum bicarbonate level was measured by an enzymatic carbonate method using a Dimension Xpand analyzer (Siemens Healthcare Diagnostics Inc., Tokyo, Japan). We defined the outcome as a decrease of 25% or more in eGFR or starting hemodialysis or peritoneal dialysis. The observation period was two years. The patients’ data were collected until they reached the outcome or changed hospital. Data from patients who changed hospitals were considered as censored observations.

### Statistical analyses

Normally distributed variables are presented as mean ± SD; otherwise, the median and interquartile range (IQR) are presented. Intergroup comparisons were performed using the chi-square test, *t*-test, and Mann–Whitney *U*-test as appropriate. Univariate linear regression analysis and multivariate linear regression analysis were carried out to identify variables that were independently associated with serum bicarbonate level by including factors that were previously selected on the basis of *p* level in univariate linear regression analysis (*p* level, 0.1 or lower). Serum bicarbonate level was defined to be low when it fell in the lower 25th percentile of serum bicarbonate level (25.5 mEq/l). Patients’ survival curves were derived by Kaplan-Meier analysis. Cox proportional hazard models were used to evaluate the relationship of serum bicarbonate level with the outcome and to compare the risk of the outcome between a group with a low serum bicarbonate level (low-bicarbonate group) and the group with a normal serum bicarbonate level (control group). We adjusted several a priori-chosen covariates sequentially. Model 2 was adjusted for patient demographics, namely, age, gender, diabetes, hypertension, and body mass index (BMI); Model 3 was adjusted for variables in model 2 and laboratory variables, namely, eGFR, serum albumin, sodium, and potassium levels, and 24-hour urine protein excretion level; Model 4 was adjusted for variables in model 3 and variables for medications, namely, RAAS inhibitor use, loop diuretic use, and sodium bicarbonate use. The results are presented here as hazard ratios (HRs) with 95% confidence interval (CI). Statistical significance was defined as *p* < 0.05. These analyses were conducted using SAS, version 9.2 (SAS, Inc., Cary, North Carolina).

## Results

One hundred and thirteen elderly CKD patients were included in the sample for analysis. Patient demographics including biochemical data are shown in Table 1. The causes of CKD were as follows: diabetic nephropathy, 38 patients, 33.6%; chronic glomerulonephritis, 70 patients, 62.0%; and others, 5 patients, 4.4%. Within two years, 46 patients (40.7%) showed a decrease of 25% in their eGFR and 10 patients (8.9%) started dialysis. Forty six patients (40.7%) reached the outcome, and the rate of reaching the outcome was 0.35 per patient-year. None of the patients died. Thirty-three patients changed hospital (29.2%): low-bicarbonate group, 9 (32.1%); control group, 24 (28.2%).

**Table 1:** Baseline characteristics of patients with low serum bicarbonate level in comparison with those of control group

	**All**	**Low-bicarbonate group**	**Control group**	***p *value**
N (%)	113	28	85	
Age (years)	70.4 ± 6.6	71.0 ± 6.9	70.2 ± 6.5	0.59
Female (%)	41 (36.3)	5 (17.9)	36 (42.4)	0.019
Diabetes mellitus (%)	38 (33.6)	5 (17.9)	33 (38.8)	0.042
Hypertension (%)	45 (39.8)	3 (10.7)	42 (49.4)	0.0003
Height (cm)	160.6 ± 8.9	164.8 ± 8.1	159.2 ± 8.7	0.005
Weight (kg)	58.3 ± 8.9	60.9 ± 9.4	57.4 ± 8.6	0.08
BMI (kg/m^2^)	22.5 ± 2.5	22.3 ± 2.2	22.6 ± 2.5	0.61
eGFR (ml/min/1.73 m^2^)	25.7 ± 13.6	15.1 ± 5.9	29.1 ± 13.6	0.0001
CKD Stage (%)				0.0001
3	40 (35.4)	1 (3.5)	39 (45.9)	
4	44 (38.9)	12 (42.9)	32 (37.7)	
5	29 (25.7)	15 (53.6)	14 (16.5)	
Albumin level (g/dl)	3.7 ± 0.3	3.7 ± 0.3	3.7 ± 0.4	0.99
Sodium level (mEq/l)	140.7 ± 2.3	140.9 ± 2.2	140.7 ± 2.4	0.63
Potassium level (mEq/l)	4.8 ± 0.5	5.0 ± 0.5	4.7 ± 0.5	0.004
Bicarbonate level (mEq/l)	27.4 ± 3.2	23.4 ± 1.8	28.8 ± 2.3	0.0001
24-hour urine protein excretion level (g/day)	0.93 ± 1.01	1.25 ± 1.19	0.83 ± 0.93	0.063
	0.52 (IQR, 0.25, 1.18)	1.04 (IQR, 0.35, 1.66)	0.44 (IQR, 0.24, 1.11)	
RAAS inhibitor use (%)	98 (86.7)	20 (71.4)	78 (91.8)	0.006
ACEI use	51 (45.1)	12 (42.9)	39 (45.9)	
ARB use	86 (76.1)	18 (64.3)	68 (80.0)	
Direct renin inhibitor use	34 (30.4)	9 (32.1)	25 (29.8)	
Loop diuretic use (%)	37 (32.7)	11 (39.3)	26 (30.6)	0.40
Sodium bicarbonate use (%)	23 (20.3)	12 (42.9)	11 (12.9)	0.0006
Dose of sodium bicarbonate (g/day)	0.54 ± 1.29	1.28 ± 2.03	0.28 ± 0.78	0.0006
	0 (IQR, 0, 0)	0 (IQR, 0, 2)	0 (IQR, 0, 0)	
Decrease of 25% or more in eGFR (%)	46 (40.7)	19 (67.9)	27 (31.8)	0.0007
Dialysis (%)	10 (8.9)	6 (21.4)	4 (4.7)	0.007
Outcome (%)	46 (40.7)	19 (67.9)	27 (31.8)	0.0007
Follow-up days (days)	449.3 ± 162.7	360.4 ± 184.6	478.6 ± 144.4	0.0007

Values are expressed as mean ± standard deviation. The levels of 24-hour urine protein excretion and dose of sodium bicarbonate are presented with median and IQR. The values are compared between the groups by the chi-square test, *t*-test, or Mann–Whitney *U*-test as appropriate.

Abbreviations: *BMI* Body mass index, *eGFR* Estimated glomerular filtration rate, *RAAS* Renin angiotensin aldosterone system, *ACEI* Angiotensin-converting enzyme inhibitor, *ARB* Angiotensin II receptor blocker, *IQR* Interquartile range; outcome, a decrease of 25% or higher in eGFR or starting dialysis.

The baseline characteristics of patients are shown in Table 1. The low-bicarbonate group and control group did not show significant differences in age, BMI, serum albumin level, sodium level, and loop diuretic use. The numbers of patients who were female, had diabetes, and had hypertension were higher in the control group. The low-bicarbonate group showed a lower eGFR, a higher potassium level, a lower bicarbonate level, a higher 24-hour urinary protein excretion level, a lower number of RAAS inhibitor users, and a larger number of sodium bicarbonate users than the control group. The dose of sodium bicarbonate in the low-bicarbonate group was from 0 to 9 g/day, and in the control group, from 0 to 4 g/day. The average dose of sodium bicarbonate in the low-bicarbonate group was higher than that in the control group. A larger number of patients reached the outcome in the low-bicarbonate group than in the control group. The rates of reaching the outcome in the low-bicarbonate group and control group were 0.69 and 0.24 per patient-year, respectively.

### Baseline serum-bicarbonate-level-associated factors

There was a correlation between serum bicarbonate level and the levels of other markers (Table 2). Serum bicarbonate level was positively associated with gender, diabetes, hypertension, and eGFR, and negatively with potassium level, 24-hour urine protein excretion level, and sodium bicarbonate use. Multivariate linear regression analysis of the variables associated with serum bicarbonate level in the univariate linear regression analysis showed that bicarbonate level was independently associated with diabetes and eGFR.

**Table 2:** Baseline serum bicarbonate level correlated with other factors

	** Univariate linear regression analysis**		** Multiple linear regression analysis**	
	**Difference in serum bicarbonate level**	***p***	**Difference in serum bicarbonate level**	***p***
Age (years)	−0.02	0.64		
Female (%)	1.16	0.06	0.80	0.13
Diabetes mellitus (%)	1.35	0.03	1.28	0.033
Hypertension (%)	1.67	0.006	0.63	0.27
BMI (kg/m^2^)	−0.045	0.73		
eGFR	0.13	0.0001	0.094	0.0001
Albumin level (g/dl)	0.44	0.61		
Sodium level (mEq/l)	0.23	0.08	0.07	0.52
Potassium level (mEq/l)	−1.66	0.0045	0.035	0.95
24-hour urine protein excretion level (g/day)	−0.58	0.049	−0.22	0.40
RAAS inhibitor (%)	1.59	0.08		
Loop diuretic use (%)	−0.64	0.31		
Sodium bicarbonate use (%)	−2.70	0.0002	−1.18	0.10

Values are expressed as differences in serum bicarbonate level (estimated parameter) and *p* values. Multiple linear regression analysis variables include gender, diabetes, hypertension, eGFR, sodium level, potassium level, 24-hour urine protein excretion level, and sodium bicarbonate use.

Abbreviations: *BMI* Body mass index, eGFR Estimated glomerular filtration rate, *RAAS* Renin angiotensin aldosterone system.

### Serum bicarbonate level and CKD progression

**Figure 1:**
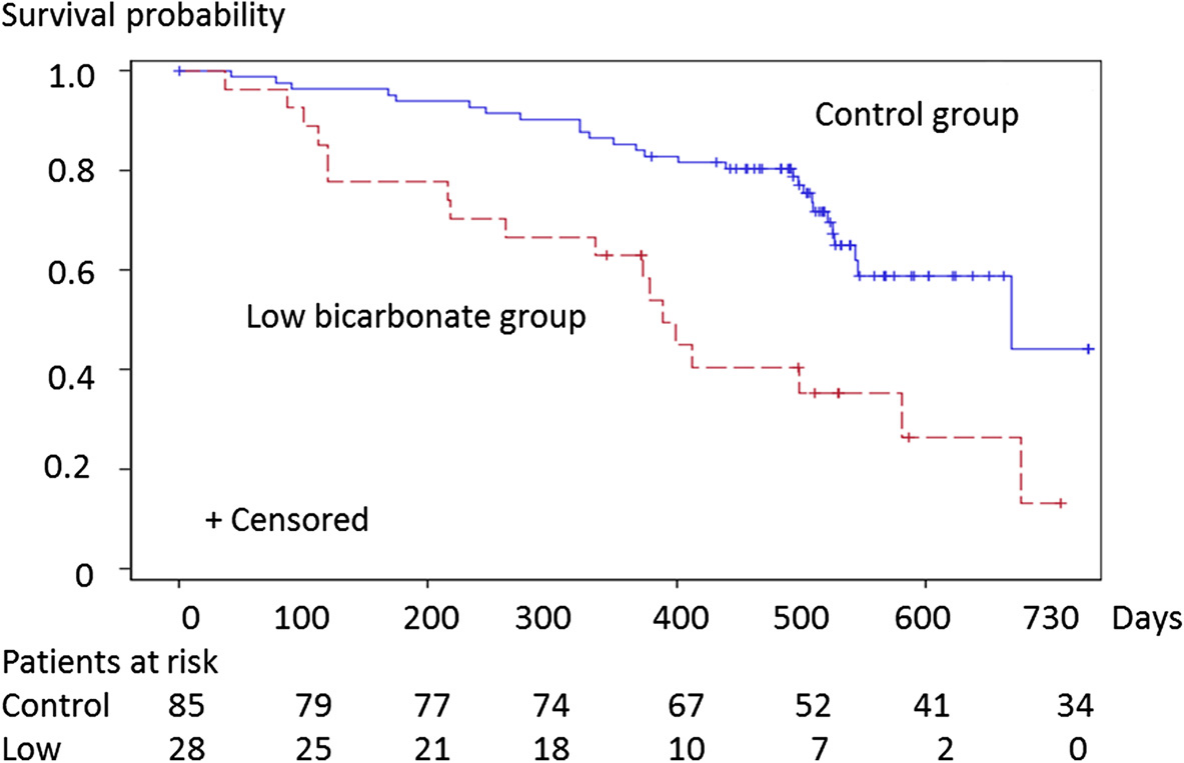
**Kaplan-Meier analysis of CKD-progression-free-survival in low-bicarbonate group compared with control group. **The numbers of patients at risk of the outcome are indicated beneath the graph. Abbreviations: CKD progression, reaching the outcome; Survival probability, probability of being CKD-progression-free.

The Kaplan-Meier analysis showed a significant difference in the outcome among the groups (Log-rank test *p* = 0.0005; Figure 1). The low-bicarbonate group was associated with a high risk of the outcome after adjustment for patient demographics, laboratory variables, and relevant medications (Table 3). A 1 mEq/l increase in serum bicarbonate level was associated with an 18.0% lower risk of the outcome (Table 3). Analysis using adjusted Cox proportional hazard models showed that this relationship was consistent; adjusted HR 0.791 [95% confidence interval (CI), 0.684-0.914] (Table 3).

**Table 3:** Hazard ratios for CKD progression according to baseline characteristics

	**Model 1**	**Model 2**	**Model 3**	**Model 4**
Serum bicarbonate level (1 mEq/l increase)	0.820 (0.735, 0.915)	0.816 (0.720, 0.924)	0.811 (0.700, 0.940)	0.791 (0.684, 0.914)
Low-bicarbonate group (ref. control group)	2.814 (1.539, 5.145)	3.310 (1.570, 6.978)	2.514 (1.068, 5.918)	3.511 (1.342, 9.186)

Values are given as HRs (95% confidence interval). Adjusted variables in four Cox proportional hazard models are as follows: Model 1, variables in univariate Cox proportional hazard model; Model 2, adjusted for variables in model 1 and variables associated with patient demographics, namely, age, gender, diabetes, hypertension, and BMI; Model 3, adjusted for variables in model 2 and variables associated with laboratory variables, namely, eGFR, serum albumin, sodium, and potassium levels, and 24-hour urine protein excretion level; Model 4, adjusted for variables in model 3 and variables associated with the use of medications, namely, RAAS inhibitors, loop diuretics, and sodium bicarbonate.

Abbreviations: *ref* Reference, *BMI* Body mass index, *eGFR* Estimated glomerular filtration rate, *RAAS* Renin angiotensin aldosterone system.

## Discussion

In this study, the serum bicarbonate levels of the elderly CKD patients were controlled within the normal range. We demonstrated that a high serum bicarbonate level was associated a low risk of CKD progression in elderly CKD patients. Our findings are consistent with previous reports that a low serum bicarbonate level is associated with CKD progression [4-7,12]. There has been no study of serum bicarbonate level in elderly CKD patients. The participants in our present study were older than those in other studies [11]. This study also showed that, although the average serum bicarbonate level of the low-bicarbonate group was controlled within the normal range in accordance with K/DOQI guidelines 2000, being in the lowest quartile of serum bicarbonate levels was associated with a high risk of CKD progression. In this analysis, a low serum bicarbonate level remained independently related to a high HR of CKD progression. These findings suggest that a high serum bicarbonate level within the normal range may prevent CKD progression in elderly CKD patients.

The optimal management of acidemia or metabolic acidosis in CKD patients, including the monitoring of serum bicarbonate level and sodium bicarbonate administration, has not been established yet. Our study showed that the patients with a serum bicarbonate level lower than 25.5 mEq/l had a high risk of CKD progression. The K/DOQI guidelines 2000 and CARI guidelines recommend that the serum bicarbonate level should be maintained at or above 22 mEq/l [16,17]. In a trial, the effect of sodium bicarbonate on CKD progression was compared between the bicarbonate-treated group and the control group [8]. The average serum bicarbonate level of the control group at baseline was 19.9 mEq/l, and the average serum bicarbonate level of the bicarbonate-treated group gradually became higher than 22 mEq/l [8]. As the lower limit, 22 mEq/l may be a useful target serum bicarbonate level in clinical practice presently.

This study showed that a high serum bicarbonate level within the normal range decreased the risk of CKD progression in elderly CKD patients. A subanalysis of the African American Study of Kidney Disease and Hypertension showed that a higher baseline serum bicarbonate level within the range from 20 to 30 mEq/l was associated with a lower risk of the composite of death, dialysis, and GFR events [7]. A retrospective cohort study of the Modification of Diet in Renal Disease Study showed that patients with serum bicarbonate levels from 26 to 40 mEq/l had a lower risk of kidney failure or death than patients with serum bicarbonate levels from 11 to 25 mEq/l [6]. However, the target serum bicarbonate level in the upper limit has not been established. A cohort study showed that patients with serum bicarbonate levels higher than 32 mEq/l showed a higher risk of death than patients with serum bicarbonate levels from 23 to 32 mEq/l [12]. The effect of a high serum bicarbonate level on mortality is related to complications such as hypokalemia, hypocalcemia, or hypomagnesemia, with resultant cardiac arrhythmias [19]. These studies suggest that, as long as serum bicarbonate level is controlled within the normal range, a serum bicarbonate level higher than the lower limit may reduce the risk of CKD progression. Thus, the upper limit of the target serum bicarbonate might be equal to the upper limit of the normal range, 32 mEq/l.

It has been reported that the capacity to excrete net endogenous acid decreases significantly with age [20]. Elderly CKD patients have a high risk of metabolic acidosis. It is necessary to regularly monitor their serum bicarbonate level to prevent metabolic acidosis. There has been no report about the mechanisms that definitely explain CKD progression in response to metabolic acidosis in not only elderly CKD patients but also young CKD patients. From studies using animal models, a few mechanisms are suggested. The increase in renal medullary ammonia level resulting from the stimulation of ammonia production by metabolic acidosis activates the alternative complement pathway and causes progressive tubulointerstitial injury [21]. New bicarbonate synthesized by the kidney in response to acidosis alkalinizes the interstitium and promotes precipitation of calcium in the kidney [22]. Increased endothelin production may mediate the tubulointerstitial injury and decrease in GFR associated with metabolic acidosis in CKD [10,23]. The mechanism underlying the renal protective effect of sodium bicarbonate in humans has not been clarified yet, either. Studies using experimental animal models of CKD suggest that alkali therapy attenuates tubulointerstitial inflammation and may slow the progression to kidney failure [21,24]. A cohort study of the African American Study of Kidney Disease and Hypertension showed that net endogenous acid production was associated with faster CKD progression in CKD patients, whose serum bicarbonate levels were within the normal range [25]. The present study showed that a low serum bicarbonate level within the normal range was independently related to a high HR of CKD progression. These results suggest that a lower serum bicarbonate level might indicate a higher net production of endogenous or exogenous acids, which might be the main cause of CKD progression. The administration of sodium bicarbonate to attain an upper limit of serum bicarbonate level within the normal range may suppress CKD progression caused by net endogenous acid production, and may prevent the subsequent events such as interstitial fibrosis that lead to CKD progression.

This study has several limitations. First, as with any observational study, we were unable to compare two groups whose characteristics were controlled. The patient distribution in CKD stages was not balanced, which might have affected the results on the CKD progression in the low-bicarbonate group. Nonetheless, this study showed that serum bicarbonate level was independently associated with CKD progression after the adjustment for patient characteristics. Second, in this study, the population included patients treated and untreated with sodium bicarbonate. The proportion of sodium bicarbonate use and the dose of sodium bicarbonate were higher in the low-bicarbonate group than in the control group. The sample size was not large enough to analyze the treated and untreated patients separately. Third, in this study, we examined 113 patients. The statistical power of this study may not be sufficient for detecting the relationship between laboratory variables and CKD progression. Fourth, the number of censored observations was high. The main reason for censoring was the change of hospital. Selection and geographical biases may have been included in this study. Fifth, acidosis-related markers such as arterial blood gas, arterial pH, and endogenous acid production were not measured. We were unable to investigate the determinants of the low serum bicarbonate levels that may contribute to CKD progression. Sixth, CKD-mineral-bone-disease-related markers, such as serum calcium, phosphate, and parathyroid hormone levels, were not measured. Therefore, we were unable to evaluate the relationship between serum bicarbonate level and bone metabolism.

## Conclusions

Our data showed a possibility that a high serum bicarbonate level within the normal range may more effectively prevent CKD progression in elderly CKD patients, and suggested that the upper limit of the target serum bicarbonate level may be that of the normal range of serum bicarbonate levels. For the prevention of CKD progression, there are issues that should be resolved: when to start the treatment with sodium bicarbonate and how much sodium bicarbonate should be administered. Clinical trials using a large sample size are required to obtain strong evidence that can help guide therapies with sodium bicarbonate.

## Abbreviations

CKD: Chronic kidney disease; GFR: Glomerular filtration rate; K/DOQI: Kidney Disease Outcomes Quality Initiative; CARI: Care of Australians with Renal Impairment; eGFR: Estimated GFR; SD: Standard deviation; RAAS: Renin–angiotensin–aldosterone system; ACEI: Angiotensin-converting enzyme inhibitor; ARBs: Angiotensin II receptor blockers; IQR: Interquartile range; Low-bicarbonate group: Group with a low serum bicarbonate level; Control group: Group with a normal serum bicarbonate level; BMI: Body mass index; HR: Hazard ratios; CI: Confidence interval.

## Competing interests

No financial or other interests to be declared.

## Authors’ contributions

Each author contributed to this manuscript. EK and MA analyzed the data and wrote the manuscript. MA and MY contributed to the statistical analysis and interpretation of the data. RK and TS contributed to the conception and design of the study and on-going progress of the study. MA, RK and TS designed and revised this study. All authors reviewed and approved the manuscript.

## Pre-publication history

The pre-publication history for this paper can be accessed here:

http://www.biomedcentral.com/1471-2369/14/4/prepub
